# Longitudinal changes of frailty in 8 years: comparisons between physical frailty and frailty index

**DOI:** 10.1186/s12877-021-02665-1

**Published:** 2021-12-18

**Authors:** An-Chun Hwang, Wei-Ju Lee, Nicole Huang, Liang-Yu Chen, Li-Ning Peng, Ming-Hsien Lin, Yiing-Jenq Chou, Liang-Kung Chen

**Affiliations:** 1 Center for Geriatrics and Gerontology, Taipei Veterans General Hospital, No. 201, Sec. 2, Shih-Pai Road, Taipei, Taiwan; 2 Department of Geriatric Medicine, School of Medicine, National Yang Ming Chiao Tung University, Taipei, Taiwan; 3 Institute of Public Health, National Yang Ming Chiao Tung University, No.115, Sec. 2, Li-Nong Street, Taipei, 112 Taiwan; 4 Aging and Health Research Center, National Yang Ming Chiao Tung University, Taipei, Taiwan; 5 Department of Family Medicine, Taipei Veterans General Hospital, Yuanshan Branch, Yilan County, Taiwan; 6 Institute of Hospital and Health Care Administration, National Yang Ming Chiao Tung University, Taipei, Taiwan; 7 Office of the Deputy Superintendent, National Yang Ming Chiao Tung University Hospital, Yilan County, Taiwan; 8 Taipei Municipal Gan-Dau Hospital, Taipei, Taiwan

**Keywords:** Frailty phenotype, Frailty index, Trajectory, Associated factors, Disability

## Abstract

**Background:**

Few studies have made longitudinal comparisons between frailty phenotype (FP) and frailty index (FI) changes. We aimed to investigate frailty status changes defined by FP and FI concurrently, and to compare the associated factors and incident disability among different combination of FI and FP trajectory groups.

**Methods:**

Data on respondents aged over 50 who completed the 1999, 2003 and 2007 Taiwan Longitudinal Study on Aging (TLSA) surveys (*n* = 2807) were excerpted. Changes of FI, FP and major time-dependent variables were constructed by group-based trajectory modeling. Logistic regression was used to investigate the associated factors and relationships with incident disability among different frailty trajectories.

**Results:**

We identified four FP trajectories – stably robust, worsened frailty, improved frailty, and stably frail and three FI trajectories – stable FI, moderate increase FI and rapid increase FI. Lower self-rated health, mobility impairment, and depressed mood were associated with unfavorable FP and FI changes (all *p* < 0.001). Regardless of FP trajectory groups, the moderate and rapid increase FI group had significantly more comorbidities than the stable FI group, and more visual, hearing, oral intake impairment, more difficulty in meeting living expenses, and poorer cognitive function in ≥65-year-olds (all *p* < 0.05). In addition, the worsened frailty, improved frailty, and stably frail groups had ORs for incident disability of 10.5, 3.0, and 13.4, respectively, compared with the stably robust group (all *p* < 0.01); the moderate and rapid increase FI groups had 8.4-fold and 77.5-fold higher risk than the stable FI group (both *p* < 0.001). When combining FI and FP trajectories, risk increased with FI trajectory steepness, independent of FP change (all *p* < 0.01 in rapid increase FI vs stable FI).

**Conclusions:**

Four FP trajectories (stably robust, worsened frailty, improved frailty, and stably frail) and three FI trajectories (stable FI, moderate increase FI and rapid increase FI) were identified. Lower self-rated health, mobility impairment, and depressed mood were associated with both unfavorable FP and FI trajectories. Nevertheless, even for individuals in stably robust or improved frailty FP groups, moderate or rapid increase in FI, either due to comorbidities, sensory impairment, cognitive deficits, or financial challenges, may still increase the risk of incident disability.

**Supplementary Information:**

The online version contains supplementary material available at 10.1186/s12877-021-02665-1.

## Introduction

Frailty is a distinct geriatric state characterized by gradually diminishing physiological reserve across different systems and increased susceptibility to physical and/or mental stressors during the aging process. This vulnerable condition independently predicts adverse outcomes among older people compared with non-frail contemporaries [1], which include reduced quality of life [2, 3], falls [4], hospitalization [5], institutionalization [6], and death [7, 8]. Given increasing recognition of frailty as a potentially reversible condition that presages disability, it has become a key target for disability prevention among rapidly aging populations.

The most widely-used frailty measurements are the phenotypic and the accumulated deficits models. The frailty phenotype (FP), measures five physical manifestations: slow gait, weak handgrip, exhaustion, weight loss, and low physical activity [9]. Frailty index (FI), scores accumulated deficits that encompass cognitive function and psychosocial aspects in addition to physical performance [10]. Unlike the fixed components of FP, researchers can instead devise their own FI according to a standard procedure [11]. Although FP and FI have both been closely linked to adverse outcomes among older people, such as mortality [7, 12] and institutionalization [6, 13], FI may predict mortality and institutionalization more precisely, while each FP level (robust, prefrail, frail) covers a broader range of risk [14].

Even without programmed intervention, frailty is a dynamic state [15]. In the SHARE study of > 15,000 middle-aged and older community-dwelling Europeans, about 40–60% of participants had unchanged FP status over 5-year follow-up, 15–20% with any frailty progressed to worse severity or died, while 30–40% had a decreased level of frailty [16, 17]. A recent meta-analysis of transitions between phenotypic frailty states supported these findings [18]. Although FI transition is not as well investigated, limited reports suggest a generally increasing trend of FI with age in population terms, while individual differences may also exist [19, 20]. Recent work has suggested changes in frailty predicts mortality independent of baseline frailty, whether defined by FP [21] or FI [22]. Notwithstanding existing literature, longitudinal comparisons between FP and FI changes remain to be explored, especially the group with discrepancy between the two definitions (e.g., favorable FP change + unfavorable FI change). In addition, regarding the factors associated with frailty changes, consideration of time-dependent association may provide more information than one-time measurement.

Hence, we studied a nationally representative cohort in Taiwan with the specific objectives to: 1) establish the frailty trajectories defined by FP and FI; 2) investigate how time-varying factors differ among combination of FI/FP trajectories; and 3) explore the relationships between FI/FP trajectories and incident disability.

## Methods

### Study population

The Taiwan Longitudinal Study on Aging (TLSA), which began in 1989, was conducted to investigate the impacts of socioeconomic factors on the health and emotional wellbeing of older adults [23]. That study recruited a nationally representative sample of community residents aged ≥50 years, who were then followed-up every 3 to 4 years. This study included participants who had completed the 1999, 2003, and 2007 TLSA surveys (*n* = 2807) to construct FP/FI trajectories, and excluded respondents who were lost to follow-up or died during this period from final analyses. Detailed information about the TLSA is provided by the Taiwan Health Promotion Administration [24]. The study was reviewed and approved by the Institutional Review Board (IRB) of Taipei Veterans General Hospital (No. 2021-05-023CC) and was conducted in accordance with the principles of the Declaration of Helsinki.

### Construction of variables

#### Frailty phenotype

Due to incomplete TLSA data on walking speed, grip strength and body weight, we used surrogate variables, as other published questionnaire-based studies have done. Exhaustion was defined by the same two questions from the Center for Epidemiologic Studies Depression Scale (CES-D) that the original FP definition used [9]. Handgrip strength and gait speed were assessed as in the Nagi questionnaire [25], by replies to survey items “difficulty in picking up or twisting using your fingers” and “can you walk 200–300 m?”, respectively, on a four-point Likert scale; respondents who answered “very difficult” and/or “can’t do it at all” were defined as having “weakness” and/or “slowness”. BMI ≤18.5 was designated as the cut-off for defining ‘body weight loss’. Physical activity was measured as the sum of the weighted score calculated from the intensity and frequency of leisure time physical activities; participants engaging in moderate-intensity activity every day, 1–2 times/week, and less than once/week, scored 4, 2, and 0.8, respectively. Those with low-intensity or sedentary activity at the same respective frequencies, scored 2, 1, and 0.4, or 1, 0.5 and 0.2. Men with summed weighted score < 3 or women scoring < 2 were categorized as having low physical activity [26]. Exhaustion, weakness, slowness, low physical activity, and weight loss (BMI ≤18.5) were re-coded by values of 1 or 0, corresponding with presence or absence of each condition, respectively. FP score was calculated as the summary score of these five conditions, ranging from 0 to 5. Supplementary Table 1 lists the original FP definition and the corresponding variables in the 1999, 2003 and 2007 TLSA questionnaires.

#### Frailty index

We designed a frailty index according to the standard procedure [11] and held a consensus meeting of geriatricians to decide which deficits to include. Similarly to the frailty index developed by Rockwood et al. [27], a total of 72 variables were selected; these encompassed: health status and comorbidities (17 items); mobility, activities of daily living (ADL) and instrumental ADL (IADL) (22 items); cognitive function (10 items); psychological status (10 items); stress (4 items) and life satisfaction (6 items); and the sensory domain (3 items). Supplementary Table 2 lists the selected variables and corresponding FI values. All variables were re-coded by values ranging from 0 to 1; 0 or 1 indicated the absence or presence of each deficit respectively, while 0.5 indicated intermediate status. Likewise, variables that were scored on four- or five-point Likert scales were assigned corresponding ordinal values (0, 0.33, 0.66, 1 in four-point Likert scale and 0, 0.25, 0.5, 0.75, 1 in five-point Likert scale), with larger values indicating more severe impairment. An individual’s FI was calculated by dividing the sum of their assessment scores for deficit items by the maximum possible score.

### Covariates

Baseline demographics included age, sex, education, marital status, urbanization of residential area, alcohol consumption (more than once/week), tobacco smoking status (current), difficulty meeting living expenses (answered “some” or “much” difficulty on a four-point Likert scale). Medical history encompassed good self-rated health (answered “very good” or “good” on a five-point Likert scale) and physician-diagnosed morbidities, including the total number documented, and hypertension, diabetes, heart disease, stroke, cancer, chronic lung disease, arthritis, peptic ulcer disease, hepatobiliary disease, hip fracture, cataract, chronic kidney disease (including renal stones) and gout. Mobility assessment included: 1) squatting; 2) standing for 15 min; 3) standing for 2 h; 4) raising both hands over head; 5) grasping objects with fingers; 6) lifting 11–12 kg; 7) running for 20–30 min; 8) walking 200–300 m; 9) climbing 2–3 flights of stairs. ADL was assessed by: 1) taking a bath; 2) dressing; 3) eating; 4) getting up from bed; 5) moving around the house; 6) toileting. IADL was evaluated by: 1) buying personal items; 2) managing money; 3) taking public transportation on one’s own; 4) doing physical work at home; 5) doing light tasks at home; 6) making phone calls. Any difficulty with each item would score 1 point. Summed scores ranged from 0 to 9 points for mobility impairment, and 0 to 6 points each for ADL and IADL impairment. Cognitive function was evaluated using the Short Portable Mental Status Questionnaire (SPMSQ) [28], which was only performed in participants ≥65 years old in 1999. Owing to discrepancies between the questionnaires administered in 1999, 2003 and 2007, we selected eight questions that were included in all three of these waves, with a higher score indicating better performance. Depressive mood was evaluated with the CES-D 10-item Likert score, in which higher scores (0–30) indicate more depressive symptoms. Sensory assessments included vision, hearing, and oral intake; answering “very poor” or “poor” on a five-point Likert scale was defined as impairment. Social participation was defined as engagement in social activities including religious, political, or trade union groups, voluntary work, or educational classes, etc.

For major time-dependent variables potentially associated with frailty change, including comorbidity, self-rated health, body mass index, mobility impairment, depressive symptoms (defined by CES-D), visual impairment, hearing impairment, oral intake difficulty, meeting living expenses, social participation, cognition (defined by SPMSQ, for age over 65), group-based trajectory modeling was applied to ascertain longitudinal groupings. For example, we identified four trajectories for number of comorbidities: 1) stable low; 2) stable moderate; 3) gradual increase; and 4) stable high. To maintain stability of the regression model given the limited sample size, we pooled the stable moderate, gradual increase, and stable high groups to facilitate comparison with the stable low group. Supplementary Table 3 shows the major time-dependent variable groupings.

### Incident disability

Disability was defined as institutionalization or needing a special caregiver at home to assist with ADL in the 1999, 2003 and 2007 TLSA surveys. Participants without disabilities in 1999 or 2003 but who had become disabled by 2007, were classed as having incident disability.

### Statistical analysis

FP and FI trajectories were constructed using group-based trajectory modeling, which assumes that participants represent a mixture of groups that each have distinctive biological trajectories [29]. To select the best model, we followed the procedures recommended by Nagin et al. [30]. To make trajectory models, we determined the number of trajectories, followed by the shapes, by testing the polynomial order including linear and quadratic terms for each group. The Bayesian Information Criterion (BIC) index was adopted to assess models’ goodness of fit. When two models were compared, a value of 2 x ΔBIC (BICcomplex model − BICsimple model) greater than 10 indicated strong evidence favoring the more complex model [31]. Moreover, each trajectory group was required to include greater than 5% of the total study sample. Thus, the best-fitting model was considered to be that with the highest BIC index and in which all trajectories met the pre-specified prevalence criterion. After classifying the trajectory groups, every participant was given posterior probabilities for each group and assigned to that with the highest probability. Nagin et al. proposed that average posterior probabilities should exceed 0.7 for each group, since this is indicative of reliability and good intragroup homogeneity [30].

To compare baseline characteristics between different FP and FI groups, analyses of variances (ANOVA) was used for continuous variables and X^2^ or Fisher exact test for categorical variables. *P*-values for trends were presented individually.

To investigate the major determinants of frailty change, we stratified the study sample into four groups based on FP and FI trajectories: Group 1) favorable FP + favorable FI change; Group 2) favorable FP + unfavorable FI change; Group 3) unfavorable FP + favorable FI change; Group 4) unfavorable FP + unfavorable FI change.

Multinominal logistic regression was applied to investigate relationships between baseline demographics, comorbidities, major time-dependent variables and FP/FI trajectories. In Model 0, we tested each variable by adjusting age, sex, education, baseline FP score and FI, while Model 1 included age, sex, education, baseline FP and FI score, and baseline demographics and comorbidities with *p*-value < 0.1 in Model 0. Model 2 was further adjusted by major time-dependent variables (Table 2 and Supplementary Table 4).

Binominal logistic regression adjusted by age, sex, education, and change of comorbidities, was used to explore the hypothetical association between FP/FI trajectory groups and incident disability in 2007.

Analyses were performed using SAS, version 9.4 (SAS Institute, Inc., Cary, NC) and SPSS, version 24.0. (Armonk, NY: IBM Corp).

## Results

### FP trajectories

**Fig. 1:**
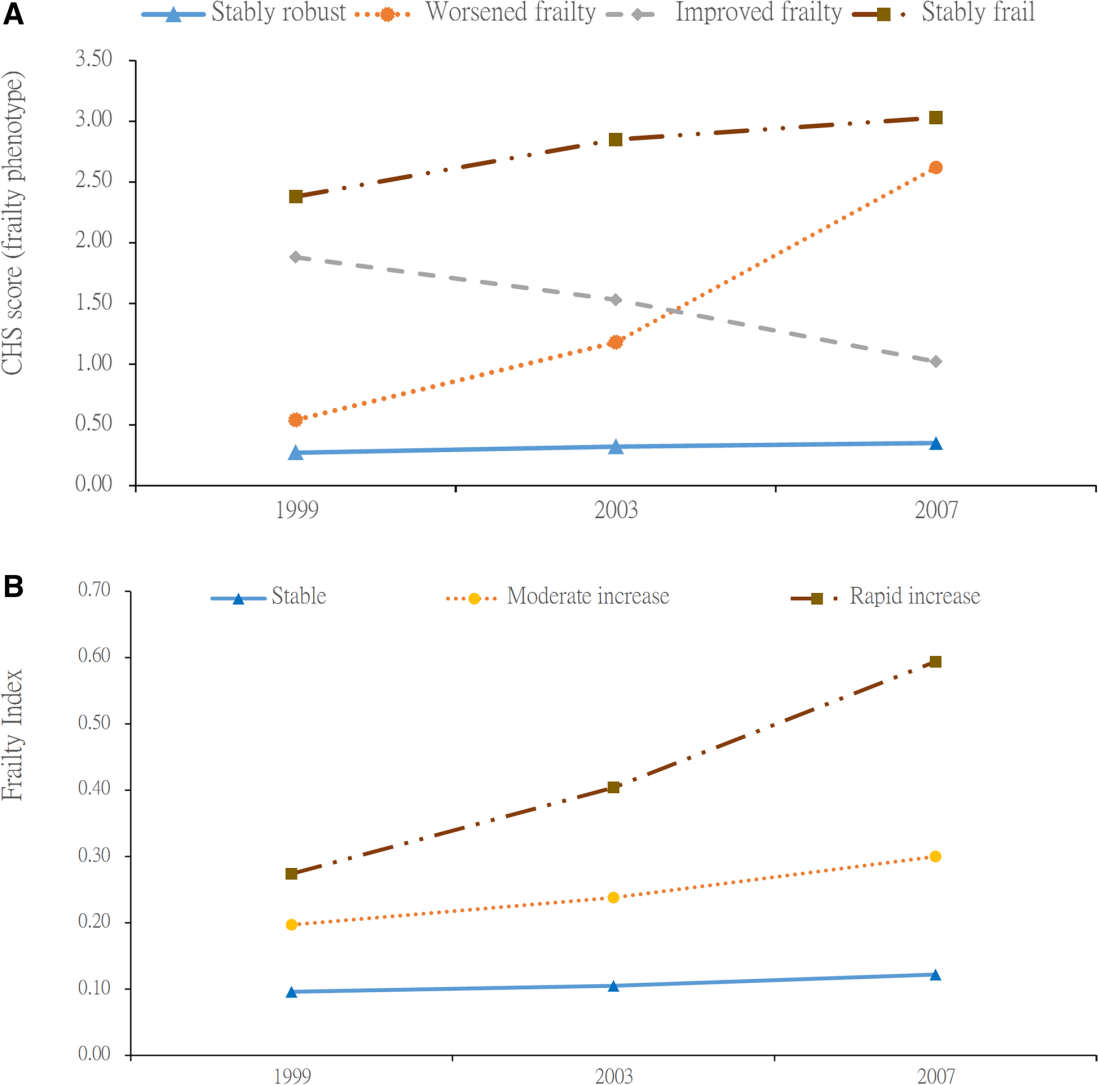
**A** Frailty phenotype transition trajectories. **B** Frailty index transition trajectories

We identified four trajectory groups for FP change (Fig. 1A, Table 1): stably robust (SR,69.8%), worsened frailty (WF, 15.7%), improved frailty (IF, 8.8%), and stably frail (SF, 5.7%). The BIC values of models with two, three, four, and five FP trajectories were − 10,885, − 10,615, − 10,379, and − 10,419, respectively. The mean posterior probabilities for the stably robust, worsened frailty, improved frailty, and stably frail groups were 0.96, 0.86, 0.84, and 0.93, respectively. In addition, the worsened frailty trajectory group model had a quadratic term, which indicated that the FP score increased even faster after the second follow-up.

**Table 1:** Comparison of baseline characteristics between different frailty phenotype and frailty index trajectories

Data show number (%) or mean ± standard deviation	Number with information available	Total	Frailty phenotype (FP)					Frailty index (FI)			
			Stably robust	Worsened frailty	Improved frailty	Stably frail	P for trend	Stable FI	Moderate increase FI	Rapid increase FI	P for trend
Number (%)	2807	2807 (100)	1960 (69.8)	440 (15.7)	248 (8.8%)	159 (5.7%)		1941 (69.1)	604 (21.5)	262 (9.3%)	
**Baseline demographics**											
Age (years)	2807	63.4 ± 8.2	61.9 ± 7.8	67.3 ± 7.9	65.2 ± 8.0	68.8 ± 7.5	< 0.001	61.6 ± 7.7	66.5 ± 7.6	69.9 ± 7.4	< 0.001
Male sex	2807	1410 (50.2)	1086 (55.4)	200 (45.5)	70 (28.2)	54 (34.0)	< 0.001	1118 (57.6)	201 (33.3)	91 (34.7)	< 0.001
Education (years)	2646	5.2 ± 4.6	6.0 ± 4.6	4.0 ± 4.1	2.7 ± 3.5	2.8 ± 3.9	< 0.001	6.2 ± 4.5	3.0 ± 3.7	2.6 ± 3.9	< 0.001
Marital status	2807						< 0.001				< 0.001
Married/cohabiting		2059 (73.4)	1523 (77.7)	277 (63.0)	163 (65.7)	96 (60.4)		1535 (79.1)	381 (63.1)	143 (54.6)	
Urbanization of residential area	2792						0.003				0.002
Large + big city		1025 (36.7)	741 (38.0)	162 (36.9)	67 (27.3)	55 (34.8)		735 (38.1)	200 (33.3)	90 (34.5)	
Small city + township		846 (30.3)	598 (30.7)	122 (27.8)	80 (32.7)	46 (29.1)		603 (31.2)	168 (28.0)	75 (28.7)	
Township (rural)		921 (33.0)	611 (31.3)	155 (35.3)	98 (40.0)	57 (36.1)		592 (30.7)	233 (38.8)	96 (36.8)	
Alcohol consumption	2804	397 (14.2)	315 (16.1)	55 (12.5)	20 (8.1)	7 (4.4)	< 0.001	321 (16.6)	58 (9.6)	18 (6.9)	< 0.001
Current smoker	2800	646 (23.1)	482 (24.7)	96 (21.9)	42 (16.9)	26 (16.4)	0.001	494 (25.5)	105 (17.4)	47 (17.9)	< 0.001
Difficulty meeting living expanses	2718	761 (28.0)	434 (22.7)	143 (33.8)	112 (46.5)	72 (51.8)	< 0.001	412 (21.7)	257 (44.1)	92 (39.0)	< 0.001
**Medical history**											
Good self-rated health	2807	1113 (39.7)	953 (48.6)	121 (27.5)	26 (10.5)	13 (8.2)	< 0.001	960 (49.5)	109 (18.0)	44 (16.8)	< 0.001
Number of morbidities	2807	1.5 ± 1.5	1.3 ± 1.3	2.0 ± 1.6	2.2 ± 1.7	2.7 ± 1.8	< 0.001	1.2 ± 1.3	2.2 ± 1.6	2.6 ± 1.8	< 0.001
Hypertension	2807	871 (31.0)	540 (27.6)	176 (40.0)	87 (35.1)	68 (42.8)	< 0.001	506 (26.1)	239 (39.6)	126 (48.1)	< 0.001
Diabetes	2807	326 (11.6)	182 (9.3)	73 (16.6)	35 (14.1)	36 (22.6)	< 0.001	162 (8.3)	96 (15.9)	68 (26.0)	< 0.001
Heart disease	2807	447 (15.9)	256 (13.1)	88 (20.0)	65 (26.2)	38 (23.9)	< 0.001	227 (11.7)	160 (26.5)	60 (22.9)	< 0.001
Stroke	2807	90 (3.2)	27 (1.4)	19 (4.3)	13 (5.2)	31 (19.5)	< 0.001	29 (1.5)	21 (3.5)	40 (15.3)	< 0.001
Cancer	2807	63 (2.2)	38 (1.9)	15 (3.4)	6 (2.4)	4 (2.5)	0.166	31 (1.6)	20 (3.3)	12 (4.6)	0.001
Chronic lung disease	2807	288 (10.3)	166 (8.5)	51 (11.6)	44 (17.7)	27 (17.0)	< 0.001	161 (8.3)	92 (15.2)	35 (13.4)	< 0.001
Arthritis	2807	551 (19.6)	313 (16.0)	98 (22.3)	91 (36.7)	49 (30.8)	< 0.001	293 (15.1)	188 (31.1)	70 (26.7)	< 0.001
Peptic ulcer disease	2807	540 (19.2)	339 (17.3)	86 (19.5)	67 (27.0)	48 (30.2)	< 0.001	337 (17.4)	146 (24.2)	57 (21.8)	0.001
Hepatobiliary disease	2807	191 (6.8)	115 (5.9)	44 (10.0)	16 (6.5)	16 (10.1)	0.035	106 (5.5)	60 (9.9)	25 (9.5)	< 0.001
Hip fracture	2807	48 (1.7)	28 (1.4)	6 (1.4)	7 (2.8)	7 (4.4)	0.002	19 (1.0)	22 (3.6)	7 (2.7)	< 0.001
Cataract	2807	606 (21.6)	343 (17.5)	131 (29.8)	71 (28.6)	61 (38.4)	< 0.001	295 (15.2)	202 (33.4)	109 (41.6)	< 0.001
Chronic kidney disease (includes stones)	2807	195 (6.9)	116 (5.9)	39 (8.9)	20 (8.1)	20 (12.6)	< 0.001	103 (5.3)	58 (9.6)	34 (13.0)	< 0.001
Gout	2807	170 (6.1)	95 (4.8)	39 (8.9)	19 (7.7)	17 (10.7)	< 0.001	97 (5.0)	45 (7.5)	28 (10.7)	< 0.001
**Functional assessments**											
Mobility impairment score (0–9)	2807	1.8 ± 2.4	1.0 ± 1.6	2.4 ± 2.2	4.1 ± 2.7	6.1 ± 2.4	< 0.001	1.0 ± 1.5	3.2 ± 2.5	4.8 ± 2.8	< 0.001
ADL impairment score (0–6)	2807	0.11 ± 0.71	0.02 ± 0.24	0.07 ± 0.51	0.20 ± 0.59	1.27 ± 2.17	< 0.001	0.01 ± 0.14	0.16 ± 0.81	0.82 ± 1.79	< 0.001
IADL impairment score (0–6)	2807	0.57 ± 1.22	0.22 ± 0.61	0.73 ± 1.22	1.35 ± 1.67	3.13 ± 2.06	< 0.001	0.18 ± 0.52	1.05 ± 1.46	2.37 ± 2.07	< 0.001
SPMSQ (age ≥ 65)	1266	7.2 ± 1.4	7.4 ± 1.2	7.0 ± 1.5	6.7 ± 1.7	6.3 ± 1.9	< 0.001	7.6 ± 0.8	6.7 ± 1.7	6.3 ± 1.9	< 0.001
CES-D-10 score (0–30)	2701	4.6 ± 5.3	3.2 ± 4.0	5.2 ± 5.3	9.9 ± 6.1	12.1 ± 6.8	< 0.001	3.1 ± 3.8	7.9 ± 6.4	8.3 ± 7.0	< 0.001
Visual impairment	2800	361 (12.9)	165 (8.4)	73 (16.6)	58 (23.4)	65 (40.9)	< 0.001	132 (6.8)	139 (23.1)	90 (34.4)	< 0.001
Hearing impairment	2792	218 (7.8)	105 (5.4)	56 (12.8)	25 (10.1)	32 (20.1)	< 0.001	95 (4.9)	65 (10.9)	58 (22.1)	< 0.001
Oral intake difficulty	2792	531 (19.0)	271 (13.9)	116 (26.5)	81 (33.1)	63 (40.6)	< 0.001	249 (12.9)	179 (29.9)	103 (39.6)	< 0.001
Any social participation	2793	1453 (52.0)	1105 (56.6)	191 (43.9)	103 (42.0)	54 (34.0)	< 0.001	1097 (56.7)	248 (41.5)	108 (41.4)	< 0.001
**Frailty assessments**											
Frailty phenotype components											
Slowness	2806	384 (13.7)	74 (3.8)	64 (14.5)	130 (52.4)	116 (73.0)	< 0.001	87 (4.5)	171 (28.3)	126 (48.1)	< 0.001
Weakness	2806	141 (5.0)	24 (1.2)	14 (3.2)	40 (16.2)	63 (39.6)	< 0.001	37 (1.9)	46 (7.6)	58 (22.2)	< 0.001
Low physical activity	2719	514 (18.9)	231 (12.2)	70 (16.4)	129 (52.9)	84 (53.5)	< 0.001	292 (15.6)	138 (23.5)	84 (32.7)	< 0.001
Exhaustion	2719	506 (18.6)	159 (8.3)	76 (17.8)	170 (70.5)	101 (73.7)	< 0.001	188 (9.9)	222 (37.9)	96 (41.0)	< 0.001
Weight loss (BMI ≤18.5)	2740	119 (4.3)	36 (1.9)	20 (4.7)	43 (17.8)	20 (13.3)	< 0.001	65 (3.4)	38 (6.6)	16 (6.5)	0.001
Summed FP score (0–5)	2807	0.59 ± 0.88	0.26 ± 0.48	0.55 ± 0.63	2.06 ± 0.75	2.42 ± 0.97	< 0.001	0.34 ± 0.61	1.02 ± 1.02	1.45 ± 1.27	< 0.001
Frailty index	2807	0.14 ± 0.10	0.11 ± 0.05	0.16 ± 0.08	0.23 ± 0.10	0.33 ± 0.13	< 0.001	0.10 ± 0.05	0.20 ± 0.09	0.28 ± 0.14	< 0.001

*ADL* activities of daily living, *IADL* instrumental activities of daily living, *SPMSQ* Short Portable Mental Status Questionnaire, *CES-D-10* Centre for Epidemiological Studies-Depression Scale, 10-item Likert score, *BMI* body mass index

During the study period, improved frailty group participants had less increase in comorbidities, better self-rated health, stable body weight, decreased depressive symptoms, and improved mobility impairment, whereas the worsened frailty group showed opposite trends (Supplementary Fig. 1).

### FI trajectories

Three trajectory groups for FI change were identified (Fig. 1B, Table 1): stable FI (SFI, 69.1%), moderate increase FI (MFI, 21.5%), and rapid increase FI (RFI, 9.3%). The BIC values of models with two, three, and four FI trajectories were − 72,410, − 69,827, and − 69,836, respectively. The mean posterior probabilities of the stable FI, moderate increase FI and rapid increase FI groups were 0.96, 0.89, and 0.96, respectively. The mean FIs in 1999 and 2007 were 0.10 and 0.12 in the stable FI group (increase 0.0025/year), 0.20 and 0.31 in the moderate increase FI group (increase 0.014/year), and 0.28 and 0.59 in the rapid increase FI group (increase 0.039/year).

### Factors associated with unfavorable FP or FI changes

Table 1 summarizes the baseline characteristics of 2807 TLSA respondents and comparisons between distinct FP and FI trajectory groups. The number and prevalence of robust (non-frail), prefrail and frail group defined by baseline frailty phenotype were 1705(60.7%), 965(34.4%), and 137(4.9%) respectively. Participants with unfavorable FI changes tended to be older, female, formally educated for fewer years, not married or cohabiting, to abstain from tobacco or alcohol, have more comorbidities and function impairment and more difficulty in meeting living expenses (*p* < 0.01); whereas better self-rated health, cognitive function, and social participation were inversely associated with the rapidity of FI increase (*p* < 0.001). In contrast, stably frail and improved frailty group were more similar in most baseline demographics, medical history and function assessment, which may contribute to the higher degree of baseline frailty status in these two groups.

Table 2 revealed the association between major factors of frailty change and favorable/unfavorable FP/FI transitions. The study sample was stratified into four groups based on FP and FI trajectories: Group 1) favorable FP (stably robust + improved frailty) + stable FI (*n* = 1839); Group 2) favorable FP (stably robust + improved frailty) + unfavorable (moderate and rapid increase) FI change (*n* = 369); Group 3) unfavorable FP (worsened frailty + stably frail) + stable FI change (*n* = 102); Group 4) unfavorable FP (worsened frailty + stably frail) + unfavorable (moderate and rapid increase) FI change (*n* = 497). Variables with *p*-value < 0.1 in Model 0 were included in a multinominal logistic regression model. There was no significant collinearity between any variables included in the fully-adjusted logistic regression model (Variance Inflation Factor all < 2) (Table 2, Supplementary Table 4).

**Table 2:** Logistic regression of frailty index (FI) *frailty phenotype (FP) trajectory groups and associated factors

**A. Demographics and Baseline morbidities**													
**FP trajectories**	**Stably robust + Improved frailty**					**Worsened frailty + Stably frail**							
**FI trajectories** (N)	**Stable** (1839)	**Moderate increase + Rapid increase** (369)				**Stable** (102)				**Moderate increase + Rapid increase** (497)			
		**Model 1**^a^		**Model 2**^b^		**Model 1**^a^		**Model 2**^b^		**Model 1**^a^		**Model 2**^b^	
		OR (95% CI)	*P*-value	OR (95% CI)	*P*-value	OR (95% CI)	*P*-value	OR (95% CI)	*P*-value	OR (95% CI)	*P*-value	OR (95% CI)	*P*-value
**Demographics**													
Age	1	1.08 (1.06,1.10)	< 0.001	1.04 (1.01,1.07)	0.003	1.05 (1.03,1.08)	< 0.001	1.01 (0.98,1.04)	0.613	1.11 (1.09,1.13)	< 0.001	1.05 (1.02,1.08)	0.001
Sex (male vs female)	1	0.6 (0.4,0.8)	0.001	0.9 (0.6,1.4)	0.761	1.2 (0.8,1.9)	0.417	2.1 (1.3,3.5)	0.003	1.0 (0.7,1.3)	0.791	1.9 (1.2,2.9)	0.004
Education (≤6 vs > 6 years)	1	1.8 (1.3,2.6)	0.001	1.3 (0.8,2.1)	0.268	1.2 (0.7,1.8)	0.483	0.7 (0.4,1.2)	0.189	1.8 (1.3,2.5)	< 0.001	1.1 (0.7,1.9)	0.650
**Baseline morbidities**													
Diabetes	1	1.5 (1.0,2.2)	0.050	1.2 (0.7,2.0)	0.497	1.0 (0.5,2.1)	0.951	1.0 (0.5,2.2)	0.979	1.8 (1.3,2.6)	0.001	1.4 (0.8,2.5)	0.233
Stroke	1	0.6 (0.2,1.4)	0.218	0.4 (0.2,1.1)	0.083	0.9 (0.2,4.1)	0.943	0.7 (0.2,3.6)	0.718	1.7 (0.8,3.3)	0.141	1.0 (0.4,2.7)	0.994
Arthritis	1	1.3 (0.9,1.7)	0.122	1.0 (0.6,1.5)	0.869	1.2 (0.7,2.1)	0.435	1.1 (0.6,1.9)	0.773	0.8 (0.6,1.1)	0.180	0.6 (0.3,0.9)	0.012
**B. Time-dependent variables**													
**FP trajectories**	**Stably robust + Improved frailty**					**Worsened frailty + Stably frail**							
**FI trajectories**	**Stable**	**Moderate increase + Rapid increase**				**Stable**				**Moderate increase + Rapid increase**			
**Time-dependent variable trajectories**				**Model 2**^b^				**Model 2**^b^				**Model 2**^b^	
Comorbidity^c^	1			2.4 (1.6,3.5)	< 0.001			0.9 (0.6,1.5)	0.734			2.4 (1.5,3.7)	< 0.001
Self-rated health^d^	1			2.2 (1.7,3.0)	< 0.001			1.7 (1.2,2.5)	0.004			3.0 (2.1,4.2)	< 0.001
Mobility impairment^e^	1			13.0 (8.5,19.8)	< 0.001			13.5 (8.0,22.5)	< 0.001			130.7 (74.4229.4)	< 0.001
CES-D-10^e^	1			13.9 (8.8,22.0)	< 0.001			7.6 (4.4,13.2)	< 0.001			34.2 (20.6,56.6)	< 0.001
Visual impairment^f^	1			2.8 (1.9,4.0)	< 0.001			1.1 (0.6,1.8)	0.853			2.1 (1.4,3.2)	< 0.001
Hearing impairment^f^	1			1.6 (1.0,2.4)	0.044			1.3 (0.8,2.3)	0.304			2.3 (1.5,3.7)	< 0.001
Oral intake difficulty^f^				1.7 (1.2,2.4)	0.005			1.4 (0.9,2.2)	0.154			2.2 (1.5,3.3)	< 0.001
Meet living expenses^f^	1			2.5 (1.6,4.0)	< 0.001			1.5 (0.8,2.7)	0.220			1.9 (1.2,3.2)	0.010
SPMSQ (age ≥ 65)^g^	1			9.7 (4.5, 20.8)	< 0.001			1.5 (0.4, 5.5)	0.578			9.2 (4.2,20.5)	< 0.001

*OR* odds ratio, *CI* confidence interval, *CES-D-10* Centre for Epidemiological Studies Depression Scale, 10-item Likert score, *SPMSQ* Short Portable Mental Status Questionnaire

^a^Model 1: (included baseline characteristics and comorbidities only) adjusted for age, sex, education, baseline FP and FI score, and variables with *p*-value < 0.1 in Model 0 (see S4 Table)

^b^Model 2: Model 1 + major time-dependent variables with p-value < 0.1 in Model 0 (see S4 Table)

^c^(stable moderate + gradual increase + stable high) V.S stable low

^d^low, middle, high

^e^(increase + stable high) vs (decrease + stable low)

^f^(stable poor + decline) vs (stable good + improve)

^g^(stable low + decrease) vs (stable high + improve)

Before adjustment for major time-dependent variables (Model 1), diabetes was the only baseline comorbidity that significantly predicted more rapidly increasing FI (moderate and rapid increase FI vs stable FI in the favorable FP change group: odds ratio [OR] = 1.5, 95% CI 1.0–2.2; *p* = 0.05. OR in the unfavorable FP change group = 1.8, 95% CI 1.3–2.6; *p* = 0.001), although this association attenuated in the fully adjusted model (Model 2). TLSA participants with arthritis/rheumatism at baseline had lower risk of unfavorable FP and FI change (OR = 0.6, 95% CI 0.3–0.9; *p* = 0.012) (Table 2).

In Model 2, age and/or male sex still predicted unfavorable FI/FP change individually. Compared with favorable FP change (stably robust + improved frailty) + stable FI group, each additional year of age was associated with 4–5% higher risk of unfavorable FI change (OR in the favorable FP + unfavorable FI change group = 1.04, 95% CI 1.01–1.07; *p* = 0.003. OR in the unfavorable FP + unfavorable FI change group = 1.05, 95% CI 1.02–1.08; *p* = 0.001), while advancing age did not have a significant effect on FP change (OR in the unfavorable FP + favorable FI change group = 1.01, 95% CI 0.98–1.04; *p* = 0.613). On the other hand, male sex was associated with about two-fold higher risk of unfavorable FP change (OR in the unfavorable FP change + favorable FI group = 2.1, 95% CI 1.3–3.5; *p* = 0.003. OR in the unfavorable FP + unfavorable FI change group = 1.9, 95% CI 1.2–2.9; *p* = 0.004), whereas there were no significant differences between males and females in FI change (OR in the favorable FP change + unfavorable FI group = 0.9, 95% CI 0.6–1.4; *p* = 0.761).

For major time-dependent variables, low self-rated health, mobility impairment, and depressive symptoms were associated with unfavorable FP and unfavorable FI changes; ORs ranged from 1.7–3.0 for low self-rated health, 13.0–130.7 for mobility impairment, and 7.6–34.2 for depressive symptoms (all *p* < 0.01). By contrast, more numerous comorbidities, sensory impairment, difficulty of meeting living expenses and poorer cognition (for age ≥ 65) were significantly related to more rapidly increasing FI, rather than unfavorable FP change. Regardless of whether the FP change was either favorable or unfavorable, the moderate + rapid increase FI group had significantly more comorbidities compared with the stable FI group (OR = 2.4 and 2.4; *p* < 0.001), and more visual impairment (OR = 2.8 and 2.1; *p* < 0.001), hearing impairment (OR = 1.6 and 2.3; *p* < 0.05), oral intake difficulty (OR = 1.7 and 2.2; *p* < 0.01), difficulty meeting living expenses (OR = 2.5 and 1.9; *p* ≤ 0.01), and poorer cognitive function (in ≥65-year-olds, OR = 9.7 and 9.2; *p* < 0.001).

### Frailty change and incident disability

**Fig. 2:**
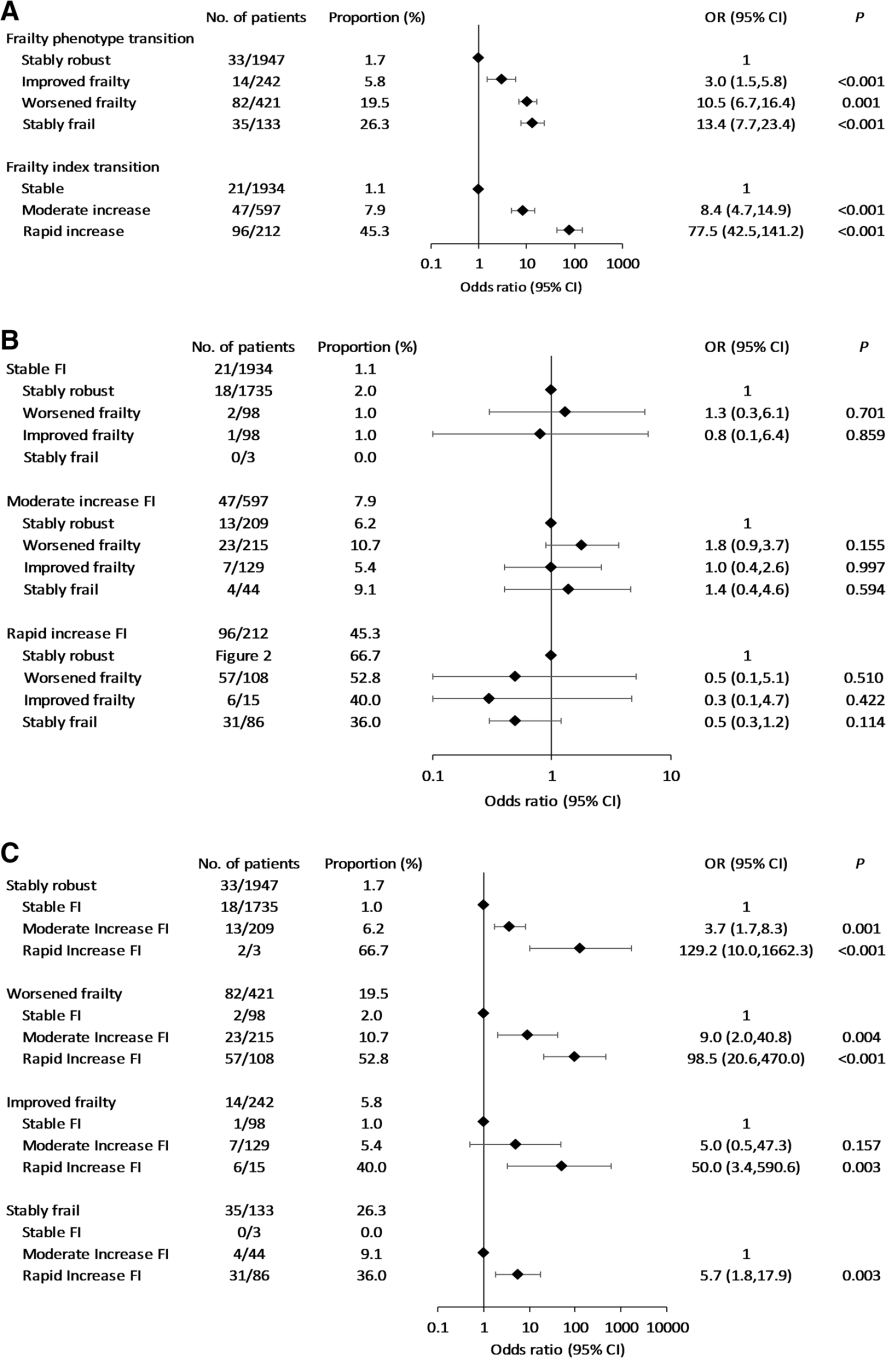
**A** Frailty transition and incident disability. **B** Frailty phenotype transition categorized by frailty index and incident disability. **C** Frailty index transition categorized by frailty phenotype and incident disability

Having excluded 64 TLSA participants who were disabled in 1999 or 2003 from the analytic cohort, 164/2743 included participants had incident disability at the 2007 follow-up. Figures 2A–C plot the strength of associations between different FP/FI trajectories and incident disability.

Compared with the stably robust FP group, the improved frailty, worsened frailty and stably frail groups had incident disability ORs of 3.0, 10.5, and 13.4, respectively (*p* ≤ 0.001). Similarly, the moderate increase FI and rapid increase FI groups had 8.4-fold and 77.5-fold higher risk compared with the stable FI group (*p* < 0.001) (Fig. 2A). However, significant associations between FP trajectory groups and incident disability disappeared when FP changes were categorized by FI trajectory groups (Fig. 2B). For example, participants with moderate increase FI had 1.0–1.8-fold higher risk of incident disability, irrespective of their FP trajectory groups (*p* > 0.15). Conversely, the risk of incident disability increased with the rapidity of FI increase across FP trajectories (Fig. 2C). Compared to participants with stable FI, those with moderate and rapid increase FI had ORs for incident disability of 3.7 and 129.2 in the stably robust FP group (*p* ≤ 0.001), 9.0 and 98.5 in the worsened frailty group (*p* < 0.01), and 5.0 and 50.0 in the improved frailty group. (*p* = 0.157 and *p* = 0.003 respectively).

## Discussion

Very few studies have explored longitudinal changes in FP and FI and made comparisons between these concurrently; our research provides interesting new insights. Among respondents to TLSA surveys from 1999 to 2007, we identified four FP change trajectories: around three quarters of this community-dwelling population had relatively unchanged frailty status over this 9-year period (stably robust and stably frail groups), 15% deteriorated (worsened frailty group), and 10% improved (improved frailty group). These proportions are commensurate with those reported in a recent meta-analysis that focused on FP transition [18]. Notably, a previous study utilizing TLSA data did not identify an improved frailty trajectory, but did find three trajectories similar to our stably robust, stably frail and worsened frailty FP groups [32]. The authors defined body weight loss using self-reported poor appetite, which might explain the difference.

In contrast with FP trajectories, the mean FIs increased in all three FI trajectory groups, with different slopes. This finding echoes the proposition that the likelihood of deficit accumulation relates to prior deficits, and improvement becomes less common over longer observation periods in general population. Nevertheless, change among individual subjects is much more heterogeneous and improvement in FI does remain possible, even in the moderate or rapid increase FI group, especially in short-term follow-up, which has been shown by previous work [33–35]. Jang et al. proposed that the one-year clinical meaningful change in frailty index was 0.02–0.05 [36], which was consistent with the change of rapid increase FI group (0.039/year). Furthermore, since frailty status fluctutes with time, whether defined by FP or FI, frailty changes predicts adverse outcome independent of baseline frailty status [37–39].

In our study, age remained a significant risk factor for moderate and rapid increase FI after adjusting for baseline demographics, comorbidities, and time-dependent variables, while the impact on FP change was insignificant. On the other hand, although women had more unfavorable FI/FP changes according to descriptive statistics, men had about two-fold risk for unfavorable FP change in the fully adjusted model, while the impact on FI change was insignificant. The impacts of age and sex on frailty change were inconsistent with previous studies, which may be due to differing definitions of frailty and inclusion of potential confounding factors [40–42].

Diabetes significantly increased the rapidity of FI increase, congruent with recent research [43], which highlights the critical role of insulin resistance and Insulin-like Growth Factor-1 in the development of frailty [44]. Attenuation of this association after adjusting for functional variables, suggests that the risk associated with diabetes may possibly be lessened by targeting deficits in mobility, mood, sensory and cognitive domains, etc. Lower risk of unfavorable FP and FI change in people with arthritis/rheumatism should be interpreted cautiously and requires further investigation to find out whether more frequent outpatient or traditional Chinese medicine use, or change to healthier lifestyle, may have been confounding factors.

The main factors associated with unfavorable FP change were lower self-rated health, mobility impairment, and depressed mood. These findings echo our previous study of a multidomain intervention against FP-defined frailty, which improved depression, gait speed and physical activity, thereby diminishing physical frailty [45]. Besides lower self-rated health, depressed mood and mobility impairment, moderate and rapid increase FI group participants had significantly disadvantageous changes in comorbidities, sensory function, cognition, and meeting living expenses, regardless of their FP trajectories. These results are congruent with the original definitions of FP and FI. FP excluded participants with cognitive impairment and focused on physical frailty, which can be considered as pre-disability syndrome [9], while FI included broader deficits that encompass comorbidities, cognition, mood, and socioeconomic circumstances [6, 46]. The differences between operational definitions of FP and FI may further account for heterogeneous relationships with incident disability. Although the likelihood of incident disability increased significantly with unfavorable FP or FI transition individually, when FP and FI trajectory groups were combined, the risk increased with unfavorable FI transition rather than FP transition. These results suggest that, even for individuals in stably robust or improved frailty FP groups, moderate or rapid rising in FI, due either to comorbidities, sensory impairment, cognitive deficits, or financial challenges, may still increase the risk of incident disability. The findings could be viewed as the longitudinal extension of previous work, indicating the risk of institutionalization and mortality increased with baseline frailty index among participants with the same frailty level defined by FP [14]. From one-time measurement to longitudinal data, there is emerging evidence revealing that the comprehensive nature in FI may make it perform better in outcome prediction in comparison with FP [38]. Further investigation combining the FI/FP trajectory groups for other adverse outcomes prediction (eg. mortality) is warranted.

We acknowledge some limitations. First, only participants who completed 1999, 2003 and 2007 TLSA surveys were included, limiting generalizability to people lost follow-up or who died consequent to high disease burden or disability. Second, the causality of relationships between incident disability in 2007 and frailty trajectories remains uncertain. Nevertheless, since trends between 1999 and 2003 and 2003–2007 were consistent in each frailty trajectory, and the impact of reverse causality was similar in FP and FI trajectories, we contend that these results are still informative; more longitudinal data will be needed to elucidate the issue. Third, FP was defined operationally by surrogate variables, which may underestimate the prevalence of frailty, although this is almost inevitable in questionnaire-based data.

## Conclusions

In conclusion, middle-aged and older people in a nationally representative sample followed four different FP trajectories (stably robust, worsened frailty, improved frailty, and stably frail) and three FI trajectories (stable FI, moderate increase FI, and rapid increase FI). Disadvantageous changes in self-rated health, mobility impairment, and depressed mood were associated with unfavorable FP and FI trajectories, while more numerous comorbidities, sensory impairment, poorer cognition (in ≥65-year-olds), and difficulty meeting living expenses may further explain acceleration of frailty index. Unfavorable transitions of both FP and FI were associated with incident disability, strongest in the rapid increase FI, followed by the moderate increase FI trajectory group.

## Supplementary Information

**Additional file 1: Supplementary Table 1.** Modified frailty phenotype and corresponding variables in the Taiwan Longitudinal Study on Aging. **Supplementary Table 2.** Frailty index variables and corresponding variables in the Taiwan Longitudinal Study on Aging. **Supplementary Table 3.** Major time-dependent variables by trajectory groups. **Supplementary Table 4.** Logistic regression between associated factors and frailty trajectory groups. **Supplementary Figure 1.** Major variables transitions among frailty phenotype trajectory groups (A–E).

## Data Availability

The datasets generated and analysed during the current study are available upon reasonable request and with permission of Ministry of Health and Welfare, Health and Welfare Data Science Center, Taiwan. https://dep.mohw.gov.tw/DOS/lp-2503-113-xCat-DOS_dc002.html

## Abbreviations

FP: Frailty phenotype
FI: Frailty index
TLSA: Taiwan Longitudinal Study on Aging
CES-D: Center for Epidemiologic Studies Depression Scale
ADL: Activities of daily living
IADL: Instrumental activities of daily living
SPMSQ: Short Portable Mental Status Questionnaire
SR: Stably robust
WF: Worsened frailty
IF: Improved frailty
SF: Stably frail
SFI: Stable frailty index
MFI: Moderate increase frailty index
RFI: Rapid increase frailty index
