# Supplementary material for: Longitudinal changes of frailty in 8 years: comparisons between physical frailty and frailty index
Source: BMC Geriatr. 2021 Dec 18;21:726. doi: 10.1186/s12877-021-02665-1 (PMC8684153; doi:10.1186/s12877-021-02665-1)
Supplement: Supplementary file 1 — Additional file 1: Supplementary Table 1. Modified frailty phenotype and corresponding variables in the Taiwan Longitudinal Study on Aging. Supplementary Table 2. Frailty index variables and corresponding variables in the Taiwan Longitudinal Study on Aging. Supplementary Table 3. Major time-dependent variables by trajectory groups. Supplementary Table 4. Logistic regression between associated factors and frailty trajectory groups. Supplementary Figure 1. Major variables transitions among frailty phenotype trajectory groups (A–E). [file 12877_2021_2665_MOESM1_ESM.docx]

**SUPPLEMENTARY MATERIALS**

**Longitudinal Changes of Frailty in 8 Years:**

**Comparisons between Physical Frailty and Frailty Index**

An-Chun Hwang, Wei-Ju Lee, Nicole Huang, Liang-Yu Chen, Li-Ning Peng, Ming-Hsien Lin,

Yiing-Jenq Chou, Liang-Kung Chen

**Contents**

| **S1 Table.** | Modified frailty phenotype and corresponding variables in the Taiwan Longitudinal Study on Aging |
| --- | --- |
| **S2 Table.** | Frailty index variables |
| **S3 Table.** | Major time-dependent variables by trajectory groups |
| **S4 Table.** | Logistic regression between associated factors and frailty trajectory groups |
| **S1 Figure.** | Major variables transitions among frailty phenotype trajectory groups (A–E) |

| **S1 Table. Modified frailty phenotype and corresponding variables in the Taiwan Longitudinal Study on Aging** | | | | |
| --- | --- | --- | --- | --- |
| **Frailty phenotype** | **Original definition in frailty phenotype** | **TLSA 1999, 2003, 2007** | **Answer options** | **Score** |
| **Exhaustion** | Self-report by two questions from CES-D: “I felt everything I did was an effort’’ or ‘‘I could not get going’’, with frequency of >3–4 days/week reported for either question. | Self-report by two questions from CES-D: “I felt everything I did was an effort’’ or ‘‘I could not get going.’’ | 0: Not at all  1: Sometimes, 1 day/week  2: Often, 2–3 days/week  3: Most of the time, ≥4 days/week | 1: option 2 or 3  0: option 0 or 1 |
| **Weakness** | Handgrip strength in the lowest 20% adjusted for sex and body mass index. | Difficulty in picking up or twisting using your fingers. | 0: No difficulty  1: Some difficulty  2: Very difficult  3: Can’t do it at all | 1: option 2 or 3  0: option 0 or 1 |
| **Slowness** | The slowest 20% of gait speed (based on time to walk 15 feet), adjusted for sex and standing height. | Can you walk 200–300 m? | 0: No difficulty  1: Some difficulty  2: Very difficult  3: Can’t do it at all | 1: option 2 or 3  0: option 0 or 1 |
| **Weight loss** | ≥10 pounds or ≥5% body weight lost unintentionally in prior year. | Body mass index ≤18.5 | 1: Yes  0: No | 1: option 1  0: option 0 |
| **Inactivity** | A weighted score of kilocalories expended/week, calculated based on self-report. The lowest 20% of physical activity was identified for each sex. | The sum of the weighted score calculated from type and frequency of leisure time activity.  **Moderate intensity**: eg. jogging, hiking, ball games.  **Low intensity**: eg. gardening, strolling, Tai Chi.  **Sedentary**: eg, watching TV, listening to the radio, reading, surfing the internet, playing Chinese chess or Mahjong, chatting with friends. | Weighted score according to type and frequency.  **Moderate intensity**  Daily: 4; 1–2 times/week: 2; <once/week: 0.8  **Low intensity**  Daily: 2; 1–2 times/week: 1; <once/week: 0.4  **Sedentary**  Daily: 1; 1–2 times/week: 0.5; <once/week: 0.2 | 1: Weighted score ≤3 for men or ≤2 for women  0: others |
| TLSA, Taiwan Longitudinal Study on Aging; CES-D, Centre for Epidemiological Studies-Depression Scale. | | | | |

| **S2 Table. Frailty index variables** | |
| --- | --- |
| **Deficit categories and component items** | **Answer** (corresponding frailty index value) |
| **I: Health status and comorbidities** (17 items) |  |
| 1. Multimorbidities (confirmed by physician diagnosis): hypertension, diabetes mellitus, heart disease, stroke, cancer, bronchitis or emphysema or asthma, arthritis or rheumatism, peptic ulcer or gastric diseases, hepatobiliary diseases, hip fracture, cataract, renal disease (including stones), gout. | ≥2 morbidities (1)  ≤1 morbidity (0) |
| 2. Hypertension | 8^a^: No (0), 0: Yes, no impact to daily life (0.33),  1: Yes, some impact on daily life (0.66),  2: Yes, much impact on daily life (1) |
| 3. Diabetes mellitus | 8: No (0), 0: Yes, no impact on daily life (0.33),  1: Yes, some impact on daily life (0.66),  2: Yes, much impact on daily life (1) |
| 4. Heart disease | 8: No (0), 0: Yes, no impact on daily life (0.33),  1: Yes, some impact on daily life (0.66),  2: Yes, much impact on daily life (1) |
| 5. Stroke | 8: No (0), 0: Yes, no impact on daily life (0.33),  1: Yes, some impact on daily life (0.66),  2: Yes, much impact on daily life (1) |
| 6. Cancer | 8: No (0), 0: Yes, no impact on daily life (0.33),  1: Yes, some impact on daily life (0.66),  2: Yes, much impact on daily life (1) |
| 7. Bronchitis or emphysema or asthma | 8: No (0), 0: Yes, no impact on daily life (0.33),  1: Yes, some impact on daily life (0.66),  2: Yes, much impact on daily life (1) |
| 8. Arthritis or rheumatism | 8: No (0), 0: Yes, no impact on daily life (0.33),  1: Yes, some impact on daily life (0.66),  2: Yes, much impact on daily life (1) |
| 9. Peptic ulcer or gastric diseases | 8: No (0), 0: Yes, no impact on daily life (0.33),  1: Yes, some impact on daily life (0.66),  2: Yes, much impact on daily life (1) |
| 10. Hepatobiliary diseases | 8: No (0), 0: Yes, no impact on daily life (0.33),  1: Yes, some impact on daily life (0.66),  2: Yes, much impact on daily life (1) |
| 11. Hip fracture | 8: No (0), 0: Yes, no impact on daily life (0.33),  1: Yes, some impact on daily life (0.66),  2: Yes, much impact on daily life (1) |
| 12. Cataract | 8: No (0), 0: Yes, no impact on daily life (0.33),  1: Yes, some impact on daily life (0.66),  2: Yes, much impact on daily life (1) |
| 13. Renal disease (including stones) | 8: No (0), 0: Yes, no impact on daily life (0.33),  1: Yes, some impact on daily life (0.66),  2: Yes, much impact on daily life (1) |
| 14. Gout | 8: No (0), 0: Yes, no impact on daily life (0.33),  1: Yes, some impact on daily life (0.66)  2: Yes, much impact on daily life (1) |
| 15. Self-rated health | 1: Very poor (1), 2: Poor (0.75), 3: Fair (0.5),  4: Good (0.25), 5: Very good (0) |
| 16. Pain | 0: None (0), 1: Mild (0.25), 2: Moderate (0.5),  3: Severe (tolerable) (0.75), 4: Very severe (intolerable) (1) |
| 17. Health status evaluated by spouse | 1: Very good (0), 2: Good (0.25), 3: Fair (0.5),  4: Poor (0.75), 5: Very poor (1) |
| **II: Physical activity, ADL and IADL** (22 items) |  |
| 1. Fall or accidental injury in past year | 0: No (0), 1: Yes (1) |
| 2. Stand for 15 minutes | 0: No difficulty (0), 1: Some difficulty (0.33),  2: Much difficulty (0.66), 3: Can’t do it at all (1) |
| 3. Stand for 2 hours | 0: No difficulty (0), 1: Some difficulty (0.33),  2: Much difficulty (0.66), 3: Can’t do it at all (1) |
| 4. Squatting | 0: No difficulty (0), 1: Some difficulty (0.33),  2: Much difficulty (0.66), 3: Can’t do it at all (1) |
| 5. Raising both hands over head | 0: No difficulty (0), 1: Some difficulty (0.33),  2: Much difficulty (0.66), 3: Can’t do it at all (1) |
| 6. Grasping objects with fingers | 0: No difficulty (0), 1: Some difficulty (0.33),  2: Much difficulty (0.66), 3: Can’t do it at all (1) |
| 7. Lifting 11–12 kilograms | 0: No difficulty (0), 1: Some difficulty (0.33),  2: Much difficulty (0.66), 3: Can’t do it at all (1) |
| 8. Run for 20–30 minutes | 0: No difficulty (0), 1: Some difficulty (0.33),  2: Much difficulty (0.66), 3: Can’t do it at all (1) |
| 9. Walking 200–300 m | 0: No difficulty (0), 1: Some difficulty (0.33),  2: Much difficulty (0.66), 3: Can’t do it at all (1) |
| 10. Climb 2–3 flights of stairs | 0: No difficulty (0), 1: Some difficulty (0.33),  2: Much difficulty (0.66), 3: Can’t do it at all (1) |
| 11. Taking Bath | 0: No difficulty (0), 1: Some difficulty (0.33),  2: Much difficulty (0.66), 3: Can’t do it at all (1) |
| 12. Dressing | 0: No difficulty (0), 1: Some difficulty (0.33),  2: Much difficulty (0.66), 3: Can’t do it at all (1) |
| 13. Eating | 0: No difficulty (0), 1: Some difficulty (0.33),  2: Much difficulty (0.66), 3: Can’t do it at all (1) |
| 14. Get up from bed; stand; sit on the chair | 0: No difficulty (0), 1: Some difficulty (0.33),  2: Much difficulty (0.66), 3: Can’t do it at all (1) |
| 15. Moving around the house | 0: No difficulty (0), 1: Some difficulty (0.33),  2: Much difficulty (0.66), 3: Can’t do it at all (1) |
| 16. Toileting | 0: No difficulty (0), 1: Some difficulty, (0.33),  2: Much difficulty (0.66), 3: Can’t do it at all (1) |
| 17. Buying personal item | 0: No difficulty (0), 1: Some difficulty (0.33),  2: Much difficulty (0.66), 3: Can’t do it at all (1) |
| 18. Managing money | 0: No difficulty (0), 1: Some difficulty (0.33),  2: Much difficulty (0.66), 3: Can’t do it at all (1) |
| 19. Riding bus/train on one’s own | 0: No difficulty (0), 1: Some difficulty (0.33),  2: Much difficulty (0.66), 3: Can’t do it at all (1) |
| 20. Doing light tasks at home | 0: No difficulty (0), 1: Some difficulty (0.33),  2: Much difficulty (0.66), 3: Can’t do it at all (1) |
| 21. Doing physical work at home | 0: No difficulty (0), 1: Some difficulty (0.33),  2: Much difficulty (0.66), 3: Can’t do it at all (1) |
| 22. Making phone calls | 0: No difficulty (0), 1: Some difficulty (0.33),  2: Much difficulty (0.66), 3: Can’t do it at all (1) |
| I**II: Cognitive domain** (10 items) |  |
| 1. Orientation to time (year) | 0: Wrong (1), 1: Right (0) |
| 2. Orientation to time (month) | 0: Wrong (1), 1: Right (0) |
| 3. Orientation to time (day) | 0: Wrong (1), 1: Right (0) |
| 4. Orientation to time (day of the week) | 0: Wrong (1), 1: Right (0) |
| 5. Recall home address | 0: Wrong (1), 1: Right (0) |
| 6. Recall mother’s family name | 0: Wrong (1), 1: Right (0) |
| 7. Recall current president’s name | 0: Wrong (1), 1: Right (0) |
| 8. Recall former president’s name | 0: Wrong (1), 1: Right (0) |
| 9. Know his/her own age | 0: Wrong (1), 1: Right (0) |
| 10. Immediate recall (10 items) | Participants answering X items, score = (1- (X/10)) |
| **IV: Psychological** (10 items) |  |
| 1. Poor appetite | 0: No (0), 1: Seldom (1 day/week) (0.33),  2: Sometimes (2–3 days/week) (0.66),  3: Usually or often (more than 4 days/week) (1) |
| 2. Everything was an effort | 0: No (0), 1: Seldom (1 day/week) (0.33),  2: Sometimes (2–3 days/week) (0.66),  3: Usually or often (more than 4 days/week) (1) |
| 3. Could not get going | 0: No (0), 1: Seldom (1 day/week) (0.33),  2: Sometimes (2–3 days/week) (0.66),  3: Usually or often (more than 4 days/week) (1) |
| 4. Insomnia | 0: No (0), 1: Seldom (1 day/week) (0.33),  2: Sometimes (2–3 days/week) (0.66),  3: Usually or often (more than 4 days/week) (1) |
| 5. Feels lonely | 0: No (0), 1: Seldom (1 day/week) (0.33),  2: Sometimes (2–3 days/week) (0.66),  3: Usually or often (more than 4 days/week) (1) |
| 6. Low mood | 0: No (0), 1: Seldom (1 day/week) (0.33),  2: Sometimes (2–3 days/week) (0.66),  3: Usually or often (more than 4 days/week) (1) |
| 7. Feel others are unfriendly | 0: No (0), 1: Seldom (1 day/week) (0.33),  2: Sometimes (2–3 days/week) (0.66),  3: Usually or often (more than 4 days/week) (1) |
| 8. Feels happy | 0: Usually or often (more than 4 days/week) (0),  1: Sometimes (2–3 days/week) (0.33),  2: Seldom (1 day/week) (0.66), 3: Never (1) |
| 9. Life goes well | 0: Usually or often (more than 4 days/week) (0),  1: Sometimes (2–3 days/week) (0.33),  2: Seldom (1 day/week) (0.66), 3: Never (1) |
| 10. Feels sad | 0: No (0), 1: Seldom (1 day/week) (0.33),  2: Sometimes (2–3 days/week) (0.66),  3: Usually or often (more than 4 days/week) (1) |
| **V: Life satisfaction, stress, and financial status** (10 items) |  |
| 1. Satisfaction with life | 0: No (1), 1: Yes (0) |
| 2. Satisfaction with what you’re doing | 0: No (1), 1: Yes (0) |
| 3. Living in a safe and secure environment | 0: No (1), 1: Yes (0) |
| 4. Satisfaction with current financial status | 1: Very good (0), 2: Good (0.25), 3: Fair (0.5),  4: Poor (0.75), 5: Very poor (1) |
| 5. Meeting living expenses | 1: No difficulty (0), 2: Fair (0.33),  3: Some difficulty (0.66), 4: Much difficulty (1) |
| 6. Stress on one’s own finances | 0: None (0), 1: Sometimes (0.5), 2: Often (1) |
| 7. Stress on one’s job | 0: None (0), 1: Sometimes (0.5), 2: Often (1) |
| 8. Stress on family member’s health, finances, or job | 0: None (0), 1: Sometimes (0.5), 2: Often (1) |
| 9. Stress on family’s relationship | 0: None (0), 1: Sometimes (0.5), 2: Often (1) |
| 10: Concerns from family and friends | 1: Very good (0), 2: Good (0.25), 3: Fair (0.5),  4: Poor (0.75), 5: Very poor (1) |
| **VI: Sensory** (3 items) |  |
| 1. Visual impairment | 0: Can see without glasses (0),  1: Can see with glasses (0.5),  2: Visual impairment (1) |
| 2. Hearing impairment | 0: Can hear without hearing aids (0),  1: Can hear with hearing aids (0.5),  2: Hearing impairment (1) |
| 3. Oral intake difficulty | 0: Can eat without denture (0),  1: Can eat with denture (0.5),  2: Poor oral intake (1) |
| ADL. activities of daily living; IADL, instrumental activities of daily living.  ^a’^8’ denotes that a responder did not have the comorbidity (according to question in the original questionnaire), meaning that the question about daily life impact was not applicable. | |

| **S3 Table. Major time-dependent variables by trajectory groups** | |
| --- | --- |
| **Variable** | **Trajectory groups** |
| Comorbidity | Stable low vs others (stable moderate + gradual increase + stable high) |
| Self-rated health | Low, middle, high |
| Body mass index | Normal vs (overweight + obese) |
| Mobility impairment | (increase + stable high) vs (decrease + stable low) |
| Depressive symptoms (CES-D-10) | (increase + stable high) vs (decrease + stable low) |
| Visual impairment | (stable poor + decline) vs (stable good + improve) |
| Hearing impairment | (stable poor + decline) vs (stable good + improve) |
| Oral intake difficulty | (stable poor + decline) vs (stable good + improve) |
| Meet living expenses | (stable poor + decline) vs (stable good + improve) |
| Social participation | (moderate + increase) vs (stable low) |
| SPMSQ (age ≥65) | (stable low + decrease) vs (stable high + improve) |
| CES-D-10, Centre for Epidemiological Studies Depression Scale, 10-item Likert score; SPMSQ, Short Portable Mental ^Status^ Questionnaire. | |

| **S4 Table. Logistic regression between associated factors and frailty trajectory groups** | | | | |
| --- | --- | --- | --- | --- |
|  | **Model 0**^a^ | **Model 1**^b^ | **Model 2**^c^ | **Collinearity** |
|  | P-value | P-value | P-value | (VIF) |
| **Baseline characteristics** |  |  |  |  |
| Age | <0.001 | <0.001 | 0.006 | 1.319 |
| Sex (female vs male) | <0.001 | 0.038 | <0.001 | 1.343 |
| Education (≤6 vs >6 years) | 0.001 | 0.006 | 0.268 | 1.430 |
| Marital status | 0.282 |  |  |  |
| Urbanization of residential area | 0.628 |  |  |  |
| Alcohol consumption | 0.321 |  |  |  |
| Current smoker | 0.462 |  |  |  |
| **Baseline morbidities** |  |  |  |  |
| Hypertension | 0.134 |  |  |  |
| Diabetes | 0.007 | 0.029 | 0.661 | 1.087 |
| Heart disease | 0.173 |  |  |  |
| Stroke | 0.008 | 0.003 | 0.069 | 1.043 |
| Cancer | 0.630 |  |  |  |
| Chronic lung disease | 0.246 |  |  |  |
| Arthritis | 0.023 | 0.060 | 0.008 | 1.103 |
| Peptic ulcer disease | 0.059 | 0.349 | 0.207 | 1.057 |
| Hepatobiliary disease | 0.281 |  |  |  |
| Hip fracture | 0.110 |  |  |  |
| Cataract | 0.370 |  |  |  |
| Chronic kidney disease | 0.515 |  |  |  |
| Gout | 0.367 |  |  |  |
| **Time-dependent variables** (by trajectory group) |  |  |  |  |
| Comorbidity (stable low vs others) | <0.001 |  | <0.001 | 1.398 |
| Self-rated health (low, middle, high) | <0.001 |  | <0.001 | 1.557 |
| Body mass index (normal vs (overweight + obese)) | 0.138 |  |  |  |
| Mobility impairment (increase + stable high) vs (decrease + stable low) | <0.001 |  | <0.001 | 1.648 |
| CES-D-10 (increase + stable high) vs (decrease + stable low) | <0.001 |  | <0.001 | 1.236 |
| Visual impairment (stable poor + decline) vs (stable good + improve) | <0.001 |  | <0.001 | 1.265 |
| Hearing impairment (stable poor + decline) vs (stable good + improve) | <0.001 |  | 0.003 | 1.209 |
| Oral intake difficulty (stable poor + decline) vs (stable good + improve) | <0.001 |  | 0.001 | 1.236 |
| Meet living expenses (stable poor + decline) vs (stable good + improve) | <0.001 |  | 0.001 | 1.113 |
| Social participation (moderate + increase) vs (stable low) | <0.001 |  | 0.144 | 1.060 |
| SPMSQ (age ≥65) (stable low + decrease) vs (stable high + improve) | <0.001 |  | <0.001 | 1.204 |
| VIF, Variance Inflation Factor; CES-D, Centre for Epidemiological Studies Depression Scale, 10-item Likert score; SPMSQ, Short Portable Mental Status Questionnaire.  ^a^Model 0: adjusted for age, sex, education, baseline frailty phenotype score and frailty index for each variable.  ^b^Model 1: included baseline characteristics and comorbidities only, adjusted for age, sex, education, baseline frailty phenotype score and frailty index and variables with p-value <0.1 in Model 0.  ^c^Model 2: Model 1 + time-dependent variables with p-value <0.1 in Model 0. | | | | |

**S1 Figure. Major variables transitions among frailty phenotype trajectory groups (A–E)**

| **A. Comorbidities** | **B. Self-rated health** |
| --- | --- |
| 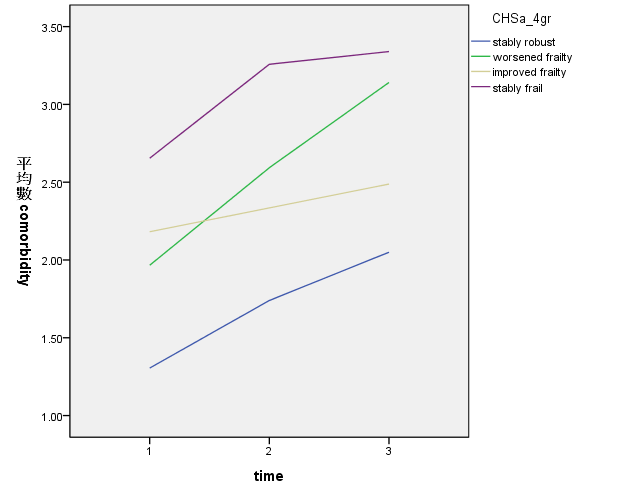   \| **FP trajectory** \| **2^nd^ TLSA wave** (2003) \| \| **3^rd^ TLSA wave** (2007) \| \| \| --- \| --- \| --- \| --- \| --- \| \| Beta (95% CI)^a^ \| P-value \| Beta (95% CI)^a^ \| P-value \| \| (1) SR \| 1 \|  \| 1 \|  \| \| (2) WF*time \| 0.19 (0.01,0.37) \| 0.035 \| 0.43 (0.24,0.62) \| <0.001 \| \| (3) IF*time \| -0.28(-0.52, -0.05) \| 0.020 \| −0.44 (−0.69,−0.18) \| 0.001 \| \| (4) SF*time \| 0.17 (−0.17,0.50) \| 0.322 \| −0.06 (−0.41,0.29) \| 0.74 \|   FP, frailty phenotype; TLSA, Taiwan Longitudinal Study on Aging; SR, stably robust; WF, worsened frailty; IF, improved frailty; SF, stably frail.  ^a^Generalized estimating equation model, includes: age, sex, education, baseline FP, baseline comorbidity, and FP trajectory group*time interaction. | 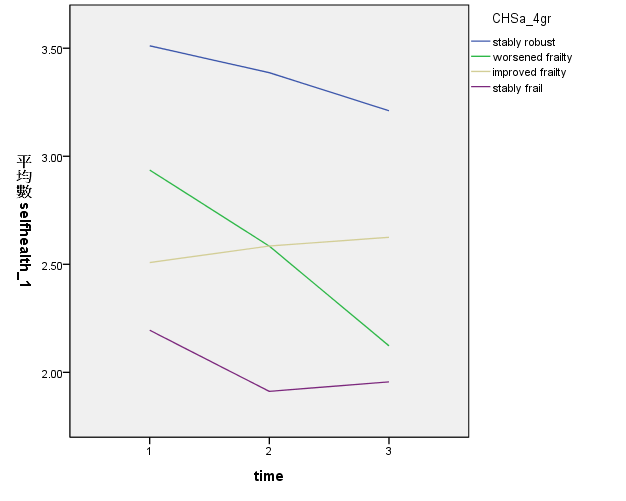   \| **FP trajectory** \| **2^nd^ TLSA wave** (2003) \| \| **3^rd^ TLSA wave** (2007) \| \| \| --- \| --- \| --- \| --- \| --- \| \| Beta (95% CI)^a^ \| P-value \| Beta (95% CI)^a^ \| P-value \| \| (1) SR \| 1 \|  \| 1 \|  \| \| (2) WF*time \| −0.23 (−0.35,−0.11) \| <0.001 \| −0.51 (−0.64,−0.38) \| <0.001 \| \| (3) IF*time \| 0.20 (0.05,0.36) \| 0.011 \| 0.42 (0.28,0.55) \| <0.001 \| \| (4) SF*time \| −0.16 (−0.33,0.02) \| 0.077 \| 0.06 (−0.11,0.23) \| 0.485 \|   FP, frailty phenotype; TLSA, Taiwan Longitudinal Study on Aging; SR, stably robust; WF, worsened frailty; IF, improved frailty; SF, stably frail.  ^a^Generalized estimating equation model, includes: age, sex, education, baseline FP, baseline self-rated health, FP trajectory group*time interaction. |

| **C. Body mass index** | **D. Depressive symptoms** (CES-D-10) |
| --- | --- |
| 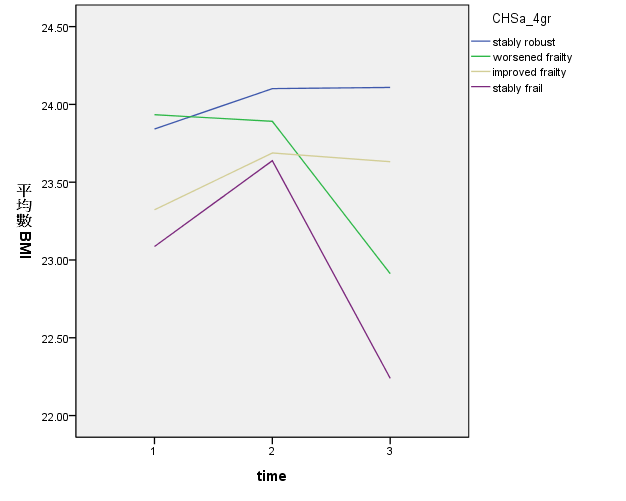   \| **FP trajectory** \| **2^nd^ TLSA wave** (2003) \| \| **3^rd^ TLSA wave** (2007) \| \| \| --- \| --- \| --- \| --- \| --- \| \| Beta (95% CI)^a^ \| P-value \| Beta (95% CI)^a^ \| P-value \| \| (1) SR \| 1 \|  \| 1 \|  \| \| (2) WF*time \| −0.33 (−0.60,−0.05) \| 0.022 \| −1.30 (−1.66,−0.94) \| <0.001 \| \| (3) IF*time \| 0.00 (−0.38,0.38) \| 1 \| 0.10 (−0.39,0.58) \| 0.691 \| \| (4) SF*time \| 0.00 (−0.68,0.69) \| 0.99 \| −1.50 (−2.32,−0.69) \| <0.001 \|   FP, frailty phenotype; TLSA, Taiwan Longitudinal Study on Aging; SR, stably robust; WF, worsened frailty; IF, improved frailty; SF, stably frail.  ^a^Generalized estimating equation model, includes: age, sex, education, baseline FP, baseline body mass index, and FP trajectory group*time interaction. | 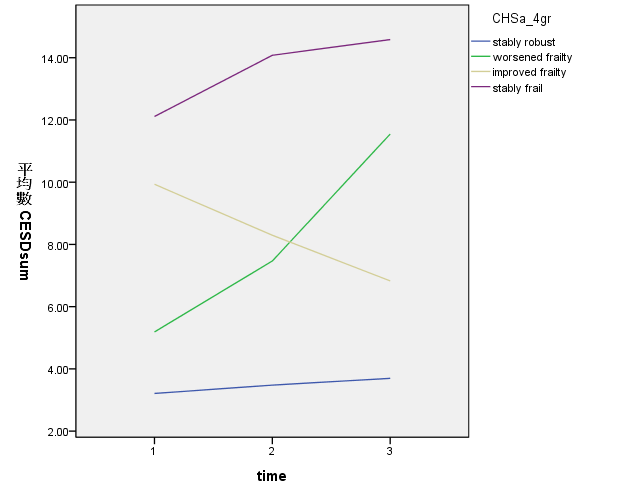   \| **FP trajectory** \| **2^nd^ TLSA wave** (2003) \| \| **3^rd^ TLSA wave** (2007) \| \| \| --- \| --- \| --- \| --- \| --- \| \| Beta (95% CI)^a^ \| P-value \| Beta (95% CI)^a^ \| P-value \| \| (1) SR \| 1 \|  \| 1 \|  \| \| (2) WF*time \| 2.01 (1.29,2.74) \| <0.001 \| 5.73 (4.88,6.58) \| <0.001 \| \| (3) IF*time \| −2.05 (−3.07,−1.03) \| <0.001 \| −3.48 (−4.47,−2.48) \| <0.001 \| \| (4) SF*time \| 1.57 (0.13,3.01) \| 0.033 \| 2.08 (0.49,3.66) \| 0.010 \|   CES-D-10, Centre for Epidemiological Studies Depression Scale, 10-item Likert score; FP, frailty phenotype; TLSA, Taiwan Longitudinal Study on Aging; SR, stably robust; WF, worsened frailty; IF, improved frailty; SF, stably frail.  ^a^Generalized estimating equation model, includes: age, sex, education, baseline FP, baseline CES-D-10, and FP trajectory group*time interaction. |

| **E. Mobility impairment** |
| --- |
| 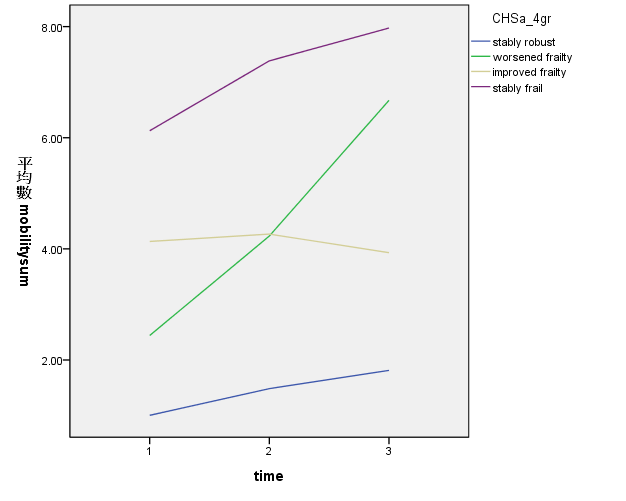   \| **FP trajectory** \| **2^nd^ TLSA wave** (2003) \| \| **3^rd^ TSLA wave** (2007) \| \| \| --- \| --- \| --- \| --- \| --- \| \| Beta (95% CI)^a^ \| P-value \| Beta (95% CI)^a^ \| P-value \| \| (1) SR \| 1 \|  \| 1 \|  \| \| (2) WF*time \| 1.31 (1.04,1.58) \| <0.001 \| 3.42 (3.15,3.70) \| <0.001 \| \| (3) IF*time \| −0.35 (−0.74,0.05) \| 0.086 \| −1.01 (−1.38,0.64) \| <0.001 \| \| (4) MF*time \| 0.78 (0.37,1.18) \| <0.001 \| 1.04 (0.61,1.47) \| <0.001 \|   FP, frailty phenotype; TLSA, Taiwan Longitudinal Study on Aging; SR, stably robust; WF, worsened frailty; IF, improved frailty; SF, stably frail.  ^a^Generalized estimating equation model, includes age, sex, education, baseline FP, baseline mobility impairment, and FP trajectory group*time interaction. |
